# Correction: Actin arginylation alters myosin engagement and F-actin patterning despite structural conservation

**DOI:** 10.1083/jcb.20240906701222026c

**Published:** 2026-02-02

**Authors:** Clyde Savio Pinto, Saskia E. Bakker, Andrejus Suchenko, Isabella M. Kolodny, Hamdi Hussain, Tomoyuki Hatano, Karuna Sampath, Krishna Chinthalapudi, Sarah M. Heissler, Masanori Mishima, Mohan Balasubramanian

Vol. 225, No. 1 | https://doi.org/10.1083/jcb.202409067| November 14, 2025

The authors have discovered that the time annotations in Figure 3 H appeared incorrectly as –1 to +1 s. The correct annotation is –3 to +3 s. Although this error does not affect the conclusions of the study, it misrepresents the timing of the event. The figure legend remains unchanged.

This error has been corrected online but appears in print and in PDFs downloaded on or before January 28, 2026. The authors apologize for any confusion this may have caused.

**Figure:**
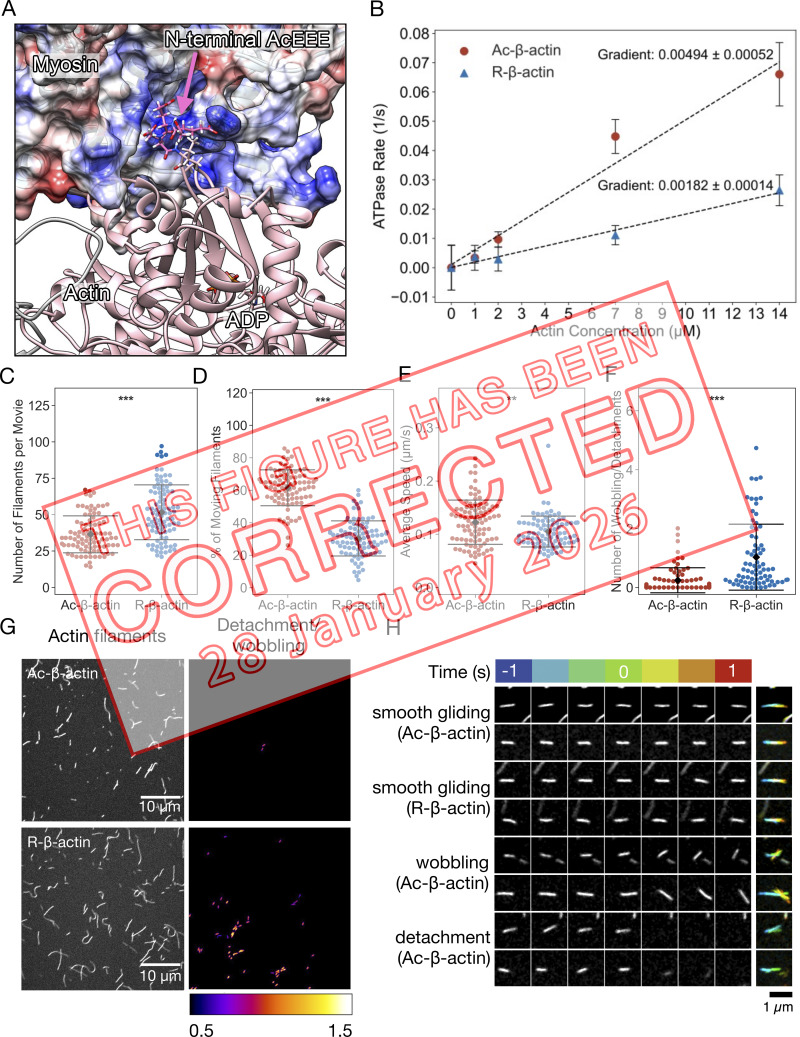


**Figure 3.**
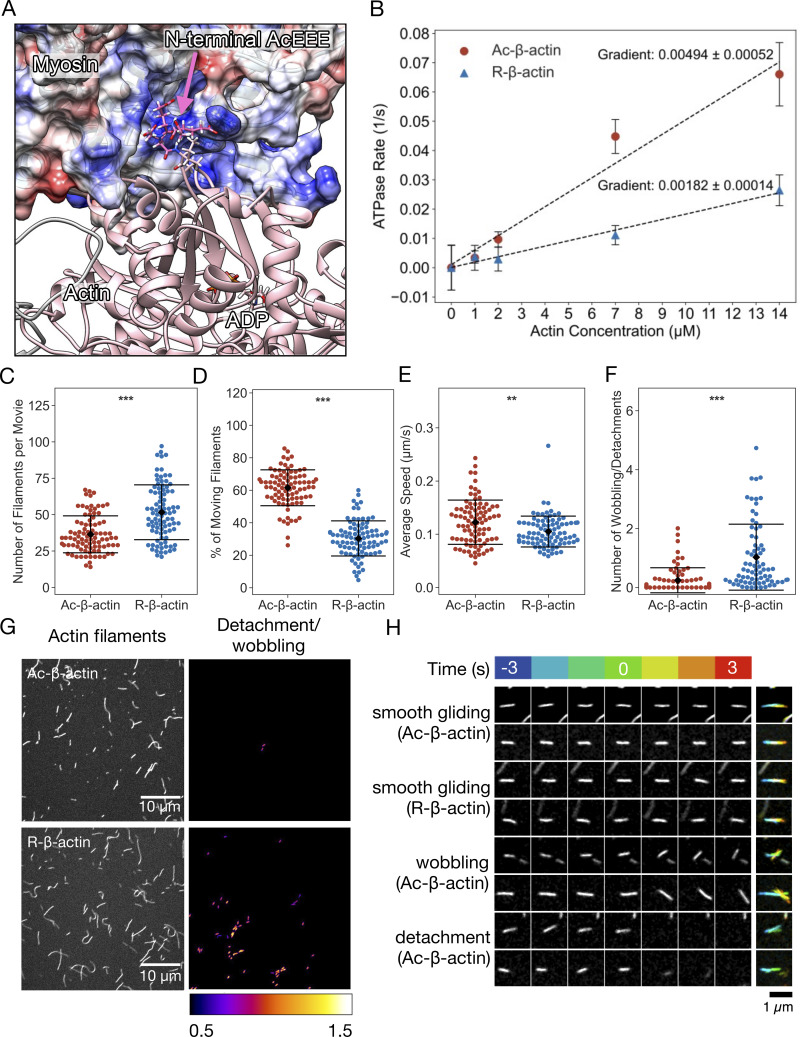
**The stability of the interaction between myosin and actin filaments is weakened by arginylation of actin. (A)** A model of the actomyosin complex, indicating the electrostatic interaction between the negatively charged actin N-terminal tail and the positively charged surface of myosin. A snapshot from a molecular dynamic simulation based on a cryo-EM structure (PDB: 5JLH [von der Ecken et al., 2016]) is displayed with the N-terminal tail of gamma-actin (AcEEEI) in magenta and the surface coulombic potential of myosin IIc in a red (0 to −10 kcal/mol) and blue (0 to +10 kcal/mol) linear scale. **(B)** The steady-state rate of ATP hydrolysis by NM myosin measured in the presence of phalloidin stabilized Ac- or R-actin filaments. **(C–F)** The quantification of the myosin-driven motility of Ac- or R-actin filaments. Each dot corresponds to an experiment or a movie of 1 min duration. Mean (diamond) and the standard error (error bars) are indicated. **(C)** Number of filaments per movies. **(D)** Percentage of the moving filaments. **(E)** Average speeds of the moving filaments per movie. **(F)** Frequency of detachment or wobbling per filament. **(G)** The surface motility assay with immobilized NM myosin and Alexa Fluor-488–conjugated phalloidin-labelled filaments of Ac- or R-β-actin. The left panels show the total filaments landed in the presence of 1 mM ATP. The right panels indicate the detachment or wobbling of filaments. **(H)** Examples of the detachment and wobbling of actin filaments. Indicated time frames before and after the detected event were shown as well as the maximum projection with temporal color coding (blue to red).

