# Supplementary material for: Actin arginylation alters myosin engagement and F-actin patterning despite structural conservation
Source: J Cell Biol. 2025 Nov 14;225(1):e202409067. doi: 10.1083/jcb.202409067 (PMC12617405; doi:10.1083/jcb.202409067)
Supplement: Table S1 — shows cryo-EM data collection, refinement, and validation statistics. [file jcb_202409067_tables1.docx]

**Supplementary table #. Cryo-EM data collection, refinement and validation statistics**

EMDB-16776, PDB 8COG

**Data collection and Processing**

Microscope FEI Titan Krios G3
Voltage (kV) 300
Camera K2
Magnification 75000
Pixel size (Å) 1.08

Exposure rate on specimen (e–/pix/sec) 0.7

Exposure time (s) 60

Total dose on specimen (e–/Å2) 42

Number of frames 39
Defocus range (μm) -0.70 to -1.50

Automation software EPU

Micrographs collected (no.) 2285

Total extracted particles (no.) 435281

**For each reconstruction**

Final particle images (no.) 41917

Point group helical
Map resolution (masked) (Å) 3.49

FSC threshold 0.143

**Model Refinement**

Initial model used (PDB code) 3J8I

Model resolution (Å) 3.49

FSC threshold 0.143

Model composition

Non-hydrogen atoms 2892

Protein residues 374

Ligands ADP, MG

R.m.s. deviations

Bond lengths (Å) (# > 4σ) 0.69 (2)

Bond angles (°) (# > 4σ) 13.3 (2)

Validation

Q-score 0.491

Clashscore 10
Poor rotamers (%) 4

Ramachandran plot

Favored (%) 94

Allowed (%) 6

Disallowed (%) 0
