# Supplementary figures and images for: Actin arginylation alters myosin engagement and F-actin patterning despite structural conservation
Source: J Cell Biol. 2025 Nov 14;225(1):e202409067. doi: 10.1083/jcb.202409067 (PMC12617405; doi:10.1083/jcb.202409067)

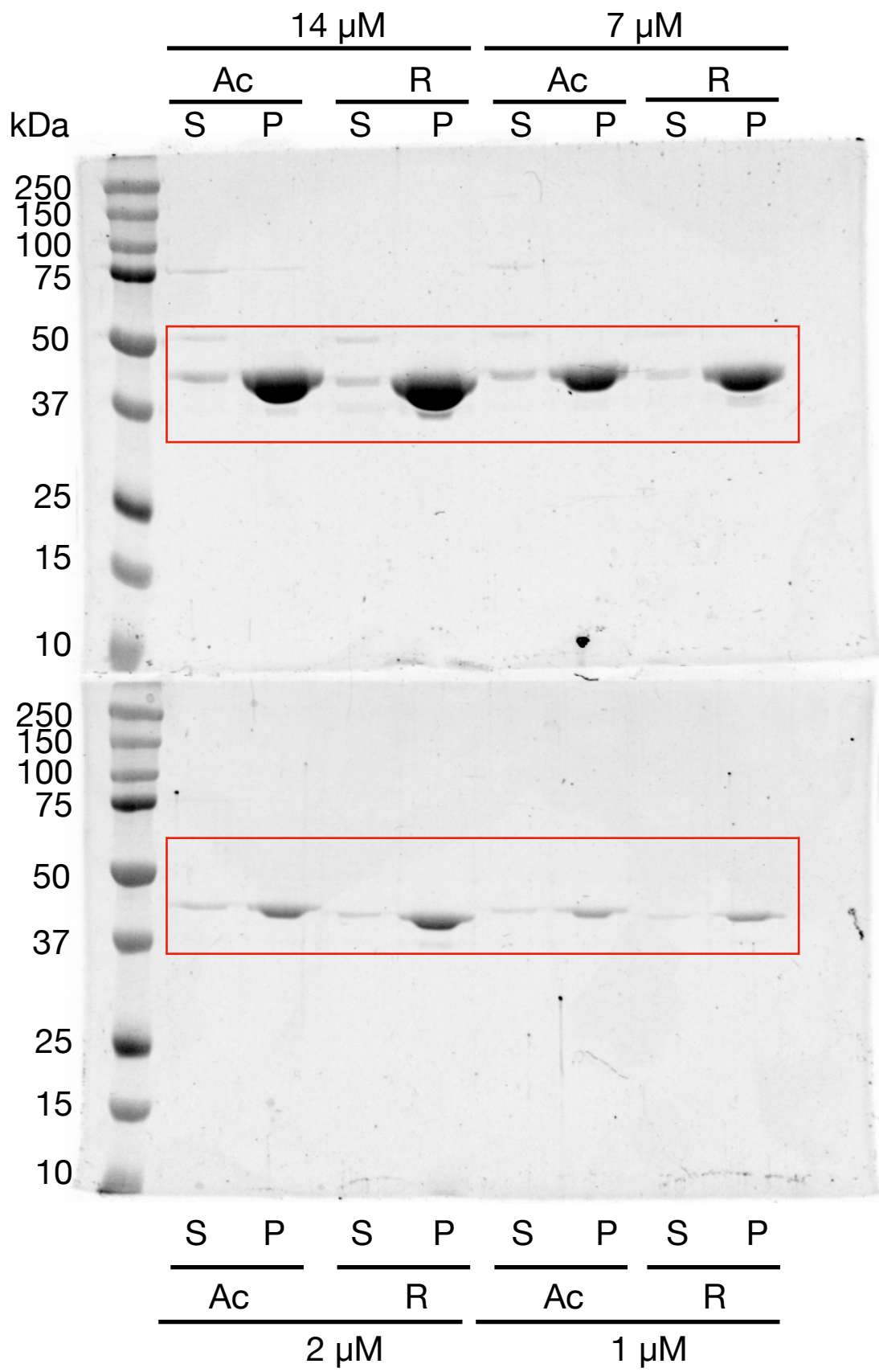

Supplement: SourceData FS1 — is the source file for Fig. S1. [file jcb_202409067_sourcedatafs1.pdf]

S4A

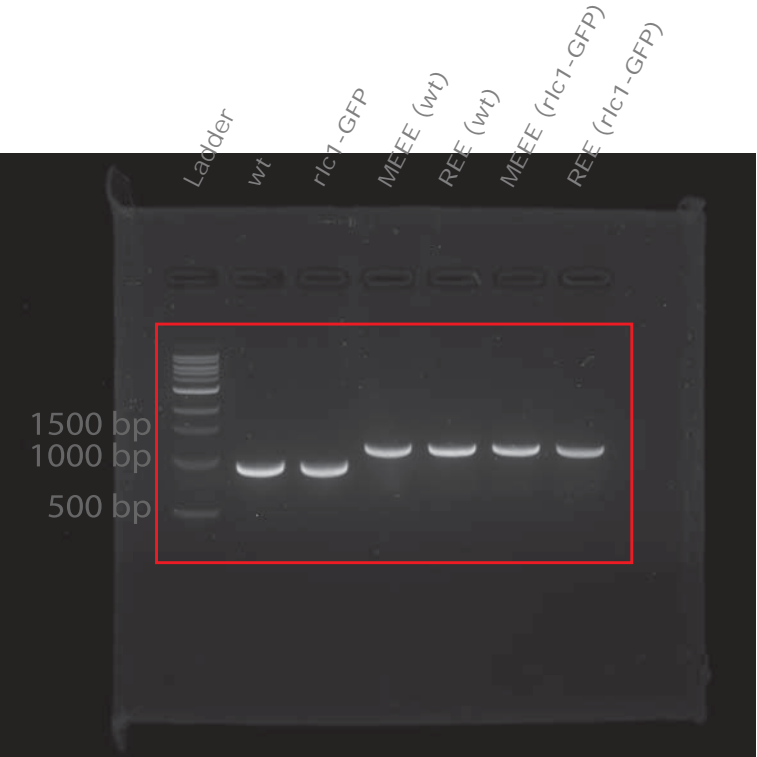

S4C

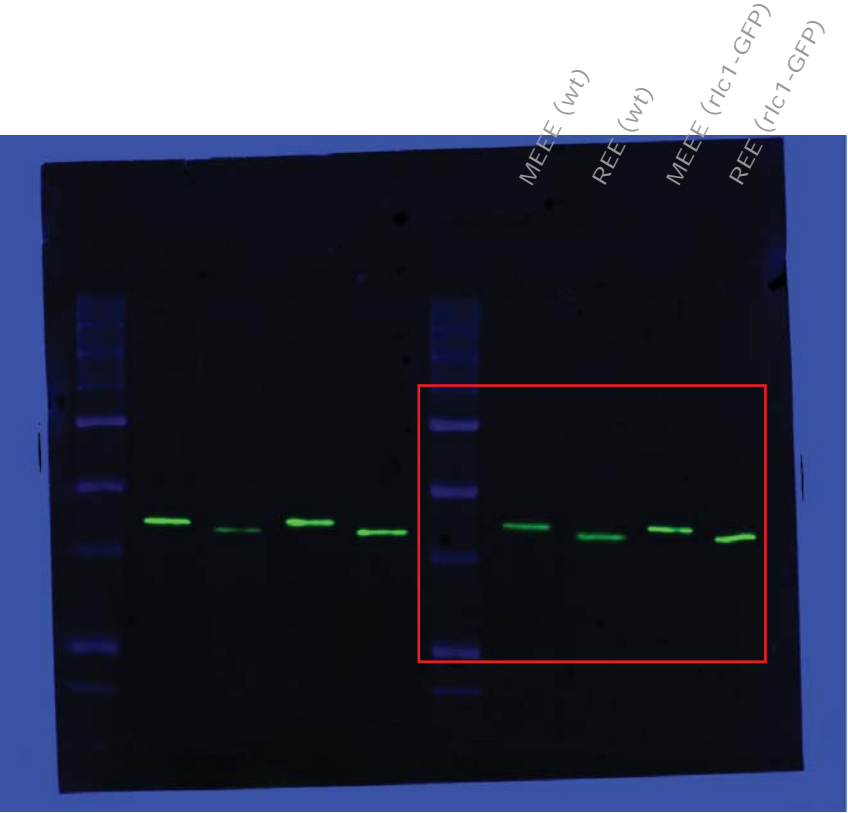

Supplement: SourceData FS4 — is the source file for Fig. S4. [file jcb_202409067_sourcedatafs4.pdf]
